# Application of Sensing Techniques to Cellular Force Measurement

**DOI:** 10.3390/s101109948

**Published:** 2010-11-05

**Authors:** Bin Li, James H.-C. Wang

**Affiliations:** 1 Department of Orthopedics, The First Affiliated Hospital of Soochow University, 188 Shizi St, Suzhou, Jiangsu 215006, China; E-Mail: binli@suda.edu.cn; 2 Orthopedic Institute, Soochow University, 708 Renmin Rd, Suzhou, Jiangsu 215007, China; 3 MechanoBiology Laboratory, Departments of Orthopaedic Surgery, Bioengineering, and Mechanical Engineering and Materials Science, University of Pittsburgh, 210 Lothrop St, BST, E1640, Pittsburgh, PA 15213, USA

**Keywords:** cell traction force, measurement, cell force monitor, micropost force sensor array, cell traction force microscopy

## Abstract

Cell traction forces (CTFs) are the forces produced by cells and exerted on extracellular matrix or an underlying substrate. CTFs function to maintain cell shape, enable cell migration, and generate and detect mechanical signals. As such, they play a vital role in many fundamental biological processes, including angiogenesis, inflammation, and wound healing. Therefore, a close examination of CTFs can enable better understanding of the cellular and molecular mechanisms of such processes. To this end, various force-sensing techniques for CTF measurement have been developed over the years. This article will provide a concise review of these sensing techniques and comment on the needs for improved force-sensing technologies for cell mechanics and biology research.

## 1. Introduction

**Figure 1.**
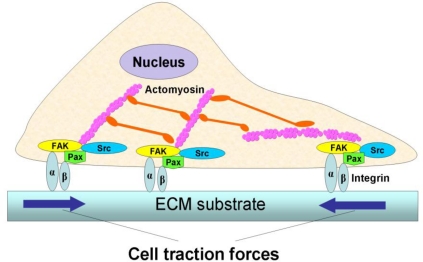
An illustration of the generation and transmission of cell traction forces (CTFs). The actomyosin interactions in the cell generate intracellular tension, which is then transmitted to an underlying substrate through focal adhesions consisting of integrins and other structural and signaling proteins (not shown). The resulting forces, shown by two arrows in opposite directions, are CTFs (adapted with permission from Figure 1 in [7]).

Cellular forces play a vital role in many fundamental biological processes. A variety of life activities, including heartbeats and body motions, rely on muscle contraction, which is ultimately determined by the intrinsic contraction of individual muscle cells. In muscle cells, cell contraction is generated by the continuous, high-speed sliding of the heads of myosin, a cellular motor protein, over actin filaments [1,2]. In non-muscle cells, a similar mechanism is used to generate intracellular tension [3]. When this intracellular tension is transmitted to the extracellular matrix (ECM) via focal adhesions (FAs), which form physical links between actin cytoskeleton and ECM, it is referred to as cell traction force (CTF) (Figure 1) [4–6].

CTFs are important in many aspects of cellular activities. Cells apply CTFs on their underlying substrates in order to enable cell migration [8,9]. Cells also use CTFs to sense the mechanical properties of their underlying substrate and adjust their adhesion and morphology. Moreover, CTFs are used to control cell shape and maintain cellular tensional homeostasis [10–12]. Therefore, CTFs are required for many fundamental biological processes, including morphogenesis, metastasis, angiogenesis, and wound healing.

In addition, CTFs are also necessary for mechano-signal transmission and transduction. Since CTFs are transmitted to ECM through FAs, which consist of diverse proteins including signaling proteins (e.g., integrins) and enzymes (e.g., kinases and phosphatases) [13], any biological, biochemical, or biomechanical stimuli acting on cells through ECM will likely cause changes in the assembly of FA proteins, the actin cytoskeleton, and actomyosin interactions These changes will in turn affect the “output” of CTFs. On the other hand, CTFs can deform the ECM network and hence produce stresses and strains in the matrix network, which in turn can modulate cellular functions such as DNA synthesis, ECM protein secretion, and even cell differentiation [6,14]. As such, CTF may be used as a useful “biophysical marker” to characterize phenotypic changes of individual cells.

In summary, a close examination of CTFs can enable better understanding of the cellular and molecular mechanisms of many important biological processes. To this end, a number of CTF-sensing techniques have been developed over the years. In this article, we will provide an overview of these cell force-sensing techniques and also illustrate their usage by giving examples of their applications.

## 2. CTF-Sensing Techniques

To date, a variety of techniques have been developed for measuring CTFs qualitatively or quantitatively. Such measurement methods can be divided into two categories: techniques for sensing the forces of a cell population and those for single cells. To measure the CTFs of a cell population, collagen-based gels with embedded cells are typically used. The gel shrinks as a result of the collective effect of cellular traction from the cell population, and the extent of such shrinkage represents the CTFs from the cell population. On the other hand, CTFs of individual cells can be detected using microscopy-based techniques, which mainly involve the use of deformable thin membranes, microfabricated structures, and hydrogels.

### 2.1. Cell population-based techniques

Cell population-based techniques are developed largely based on a cell-populated collagen gel (CPCG) model or its derivatives. CPCG was originally developed as an engineered skin graft substitute for burn patients and was, in fact, specially termed fibroblast-populated collagen lattice (FPCL) [15]. CPCG has been widely used as an *in vitro* model for measuring cell contractility [16]. In CPCG approaches, gels are used as a “force-sensing device”, with which contractile forces of cells are indirectly measured by changes in gel volume or area or directly measured with force-gauges [17–20]. As cellular contraction is directly related to CTFs, such methods are an indirect means to measure CTFs [21].

#### 2.1.1. Gel geometric change-based CPCG models

Techniques that monitor the geometric changes of collagen gel during culture represent a classical and simple approach to measuring cellular contraction semi-quantitatively. According to the anchorage status of CPCG to the substrate during measurement, three types of CPCG models have been developed. In free-floating CPCG (FF-CPCG), the gel floats in cell culture medium without any constraints, and as a result, isotonic contraction is created, resulting in a decrease in gel diameter. In tethered CPCG (T-CPCG), the gel is tightly attached to a substrate and so cannot move or relax. This results in isometric contraction of the gel, leading to a decrease in the height of the tethered gel but not in its diameter. In tethered-delayed-released CPCG (TDR-CPCG), the cell-imbedded gel is first attached to a substrate for a certain period of time to allow tension development within the gel. The gel is then released and starts to contract isotonically as a result of unconstrained cellular contraction [21,22].

**Figure 2.**
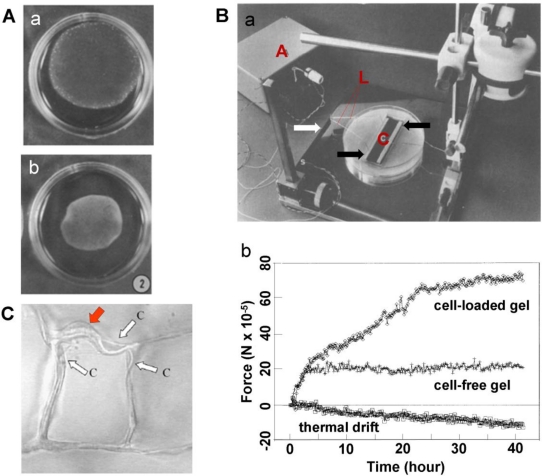
The cellular contractile forces sensed using a CPCG model. **A. (a)** Collagen gel contracts and exhibits a decrease in size; **(b)** the collagen gel further contracts, and its size is further reduced (adapted with permission from Figure 2 in [23]). **B. (a)** An experimental set-up for culture force monitor. Microporous polyethylene bars (indicated by the black arrows) are attached to a collagen gel and float in culture medium. The strain gauge beam is marked with a white arrow. The beam and a bar are connected using an A-shape frame (L) made from stainless steel suture wire. The amplifier (A) is also shown. **(b)** Cell forces change with time (adapted with permission from Figures 1–3 in [19]). **C.** Within a collagen-GAG foam-like gel, an individual dermal fibroblast (red arrow) elongated and deformed several surrounding struts (white arrows) in the scaffold (adapted with permission from Figure 1 in [26]).

The CPCG-based approaches measure cellular forces by quantifying collagen gel shrinkage [23,24]. During culture, the size of the CPCG is progressively reduced to balance the cells-generated contraction (Figure 2A). Therefore, measuring the reduction in the geometric features (such as diameter of sphere-shaped gels, area or length of rectangular gels of FF-CPCG and TDR-CPCG, and height of T-CPCG) provides indirect quantification of cellular contractility [17,22,25].

A drawback of the geometry-dependent measurement methods is that they provide only a gross estimate of cellular contractility due to large variation and instability of gel geometry during culture. An improved method involves using a collagen-GAG foam-like gel to measure the contractile force of embedded cells [26]. In addition to calculating the averaged contractile force of a cell population from gross gel deformation, the open-cell structure of this gel also allows for determination of contraction by individual cells using conventional column buckling relationships. When cells are grown in the scaffold they deform their surrounding struts. By determining the deformation of struts, cell-mediated contractile force can be calculated according to Euler’s buckling relation and the hydrostatic compression end restraint. This approach extends previous methods for analyzing cell buckling of two-dimensional (2-D) substrates to three-dimensional (3-D) constructs and can therefore be used to estimate the contractile forces of individual cells in 3-D conditions. Such a technique is significant as it can be used for cell mechanics studies using porous tissue engineering scaffolds that are structurally similar to low-density, open-cell foams.

#### 2.1.2. Culture force monitor

While conceptually simple, geometric change-based CPCG models lack sufficient sensitivity when the cellular contractile forces are relatively small. Directly sensing the force in CPCG with reasonably high sensitivity, therefore, represents a more favorable approach. This is achieved by attaching strain gauges to the CPCG to continuously track the changes in the strain or stress of the gel. Because of the ability to directly sense cellular forces, such a technique is specially termed culture force monitor (CFM). Depending on the sensitivity of the strain gauges, which function as force sensors, the CFM technique can measure small cellular contractile forces when geometric changes in gel are hardly detectable.

In a CFM system, the collagen gel can either float in medium or be tethered to an underlying substrate. In the setup developed by Delvoye *et al.*, a floating collagen gel was restrained at both ends by curing the collagen on immobilized glass rods, one of which was connected to a strain gauge [17]. In tethered collagen gels, CFM measures the isometric contraction generated within the gel with minimal changes in its dimensions [27,28]. In a typical CFM system, two bars attached to a collagen gel are connected to a central measuring beam, to which strain gauges are attached in a full bridge network to give maximum sensitivity [19] (Figure 2B). Compared to the geometric change-based CPCG methods, the sensitivity of CFM is markedly higher. For example, a displacement as small as 0.5 mm, barely measurable using geometric change-based method [19], could be detected. As a matter of fact, CFM was able to sense cell-mediated force generation during the initial stages of contraction, which is otherwise too small to be detected [19]. In addition, different cell populations could be distinguished by comparing the contraction profiles of the cells. For example, using CFM measurement, ocular fibroblasts were shown to exhibit a marked difference in their contraction profiles—corneal fibroblasts generated the strongest contraction while scleral fibroblasts produced the weakest [29]. Therefore, such a technique may serve as a useful biophysical cell profiling tool to identify cells [30].

In light of the sensitivity of CFM technique, efforts have been made to improve its efficiency over the past two decades. For example, multi-station CFM systems consisting of four vertical cantilever beams with semiconductor strain gages have been developed [31–33]. Such CFM systems are able to test multiple samples simultaneously and facilitate statistical design and analysis of experiments. In addition, a dynamic CFM (D-CFM) system has also been developed. By using computer-controlled linear actuators, this system is able to apply precise motion waveforms to multiple CPCGs independently and detect the differences in force patterns [33]. Thus, such a system may facilitate the study of the effect of dynamic mechanical loads on cells, ECM remodeling, and cell-matrix interactions. A combination of CFM and time lapse reflection microscopy, a simultaneous imaging and micro-culture force monitor (SIM-CFM) system, has been developed to measure the mechanical strain generated during matrix contraction while simultaneously recording cell and matrix behaviors [34].

### 2.2. Single cell-based techniques

Cell population-based techniques are able to provide an estimation of the “averaged” forces of a group of cells. They also have the advantage that the measured forces of cells embedded in a 3-D matrix are more physiologically relevant to cells *in vivo* that reside in a 3-D tissue environment. However, these methods do not measure CTFs *per se* [35]. Meanwhile, cells are heterogeneous and the forces that they generate vary in a wide range. Therefore, sensing forces generated by individual cells is of special importance, especially in terms of relating cell behaviors to the mechanical characteristics of cells.

To this end, a few single cell-based techniques for sensing or measuring CTFs have been developed using optical or microelectronic approaches. All these techniques can determine CTFs of individual cells at sub-cellular level, meaning that they can “map” the CTFs of a single cell. A common feature of these techniques is that they all involve the use of deformable substrates that are compliant enough to sense the tiny forces generated by a single cell. Such substrates are either continuum sheets, including wrinkle-able thin silicone membranes [4,36,37] and fluorescent beads-embedded polyacrylamide gels [38–40], or non-continuous substrates, including microfabricated cantilever arrays [41] and micropost arrays [42–46].

#### 2.2.1. Ultra-thin silicone membranes

**Figure 3.**
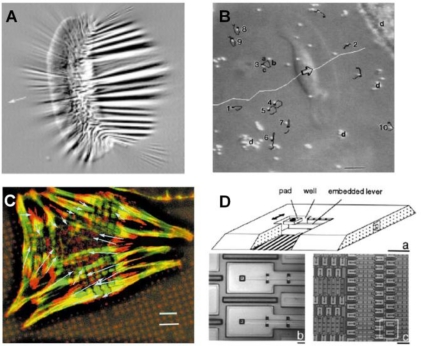
Four examples of sensing techniques for measuring CTFs. **A.** Thin silicone membrane. Note that a large number of wrinkles are created on the membrane by CTFs of a single cell (reproduced with permission from Figure 1 in [12]). **B.** Micro-beads-embedded elastic membrane (reproduced with permission from Figure 7 in [37]). **C.** Micropatterned silicone membrane with an array of fluorescent micro-dots (reproduced with permission from Figure 4 in [4]). **D.** Microfabricated cantilever array (adapted with permission from Figure 1 in [41]).

Early studies of cell mechanics used thin silicone membranes to demonstrate that fibroblasts created wrinkles on the membrane through CTFs (Figure 3A) [6,36,37]. While such a thin silicone membrane is able to “sense” small CTFs, this approach is only qualitative. This technique was further improved by estimating CTFs through applying a flexible micro-needle of known stiffness to reverse the wrinkles generated by the cell [47]. However, due to the fact that wrinkling is a nonlinear problem, there is currently no known mathematical method to accurately predict the wrinkles caused by a complex, non-isotropic CTF field. As a result, this approach cannot determine the absolute values of CTF.

Alternatively, techniques using micro-beads-embedded elastic membranes were developed in an attempt to track the displacements of micro-beads, which were used as position markers, and thereby determine CTFs at certain locations [48,49] (Figure 3B). Further, the use of micropatterned elastomer represents an important innovation, in which the surface of an elastic ultra-thin membrane is decorated with an array of fluorescent micro-dots of a fraction of a micron in height [4] (Figure 3C). By using the micro-dots as markers to determine substrate deformations, the CTFs at each FA of the cell can be determined using elasticity theory [7].

While the use of the micropatterned substrate simplifies the determination of CTFs-induced displacement fields, the stiffness of silicone membranes cannot be adjusted low enough to sense small deformations [50]. Therefore, these methods generally lack sufficient resolution in measuring small CTFs. Nevertheless, an advantage of such membrane-based techniques is that they can be easily integrated into micro-chips for biosensor applications [51].

#### 2.2.2. Microfabricated cantilever array

A technique based on a micro-machined array of cantilever beams has been developed, partially for avoiding the complicated computation associated with wrinkling membranes and for determining the absolute values of forces generated by a cell (Figure 3D) [41]. In this method, cells were cultured on pads underlined with cantilever beams of known stiffness. When the cell applies CTFs and bends a cantilever beam, a force-sensing unit, the extent of bending is recorded and the CTF is then determined accordingly. Such a technique can reliably determine the CTFs of an individual cell. However, it can only determine the CTFs in one direction. Moreover, the fabrication of the device is complicated and costly. The resolution of this technique is not satisfactory either, due to the intrinsic limitation of the fabrication process.

#### 2.2.3. Micropost force sensor array

**Figure 4.**
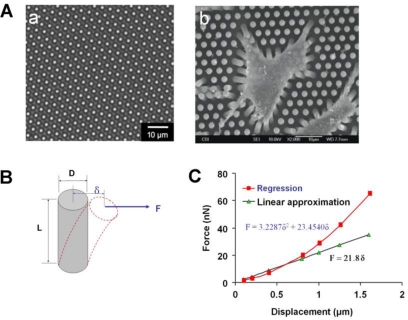
Micropost sensor array. **A. (a)** Microposts that are distributed on a silicone elastomer base. **(b)** Bending of microposts by a cell residing on top of the microposts (adapted with permission from Figures 5 and 6 in [42]). **B.** A single micropost. It functions as a cantilever beam and deflects under a pointed force (F), which represents a CTF. **C.** CTF calculation. When the deflection is small, the beam theory can be used to obtain a linear relationship between CTF and the deflection, or the displacement at the top of the micropost. When CTF is large, a non-linear relation between CTF and displacement has to be used for an accurate determination of the CTF.

To overcome the limitations associated with cantilever arrays, CTF measurement techniques based on micropost force sensor arrays (MFSAs) have been developed in recent years [42–44,52,53]. In a MFSA, each micropost functions as an individual force-sensing unit and independently senses the CTFs locally applied by a cell. Specifically, when a cell adheres to the microposts, it exerts tensile forces at the top of microposts and causes lateral deflection in them (Figure 4A) [42]. Once the deflections of the microposts are obtained by imaging analysis, the CTFs can be determined based on the beam deflection-force relationship that has been calibrated, or be calculated according to well-established beam theory [54] (Figure 4B). However, such a linear relationship between deflection and force in the beam theory is no longer valid if the deflection exceeds a certain level, when regression approximation must be applied (Figure 4C).

Compared to micro-cantilevers which can only determine CTFs in a single direction [41], microposts in an array can detect CTFs in all directions. Therefore, MFSA technology is especially useful in mapping the forces during cell migration [44,55]. Another advantage of MFSA is that the microposts can also be used to apply localized mechanical forces to a cell under a magnetic field if magnetic nano-wires are embedded inside the microposts [56]. Similarly, taking advantage of the periodicity of the micropost array, an optical Moire-based traction forces mapping technique was explored by acquiring the diffracted Moire fringe pattern of the array instead of tracking the displacements of individual microposts [53]. This method may potentially determine CTFs at a higher resolution because of the magnification effect of Moire fringe pattern as well as lack of need to track or visualize individual microposts.

#### 2.2.4. Cell traction force microscopy

**Figure 5.**
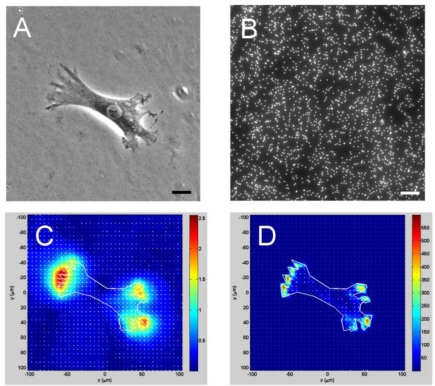
An illustration of CTFM procedures with an application example. A cell resides on an elastic substrate typically made of polyacrylamide gel **A**. The cell exerts CTFs on the underlying gel and so deforms it. The gel deformation is measured by displacements of fluorescent beads embedded in the gel **B**. By imaging analysis with a set of computer algorithms, a displacement map can be obtained **C**. From the displacements, a CTF map that represents CTF distribution beneath the cell can be obtained by solving an inverse problem (*i.e.*, “converting” displacements to CTFs) using elasticity theory or finite element method **D**.

One shortcoming of the MFSA technique is that it can only measure CTFs at predetermined, discreet points. A further advancement in CTF measurement is cell traction force microscopy (CTFM), which can determine CTFs in the entire cell spreading area [7,49,57,58] (Figure 5). CTFM represents a combination of experimental and computational approaches for CTF determination. It relies both on optical tracking of fluorescent microbeads embedded in an elastic substrate to determine deformation of substrate and subsequently on elasticity theory to determine CTFs by computation. Therefore, in essence, the elastic substrate in CTFM functions as a “sensor” of substrate deformations produced by CTFs, and the elastic theory is basically used to “convert” the substrate deformations into CTFs.

A general scheme of CTFM is as follows: first, fluorescent microbeads-embedded elastic hydrogels made of polyacrylamide or gelatin [12], or silicone membranes that are surface-coated with fluorescent beads [49], are used as cell culture substrates. When cultured on a gel, cells generate CTFs, deform the gel, and cause the embedded beads which serve as position markers to dislocate. A pair of images of the microbeads, referred to as “force-loaded” and “null-force” images, respectively, is taken using fluorescence microscope. The “force-loaded” image is taken while the adherent cells remain on the gels, whereas the “null-force” image is taken after the cells have been removed. Two steps are involved in deriving CTFs from this pair of images. The first step is to solve an image registration problem in which beads from the two images are matched and thus gel displacement field is derived. By assuming that image intensity change is a result of substrate surface movement caused by CTFs, several approaches have been used for CTF image registration, including particle tracking velocimetry (PTV) [59], particle image velocimetry (PIV) [39,57,59], feature-based registration [60], and correlation-based PTV [59]. The second step is to solve an inverse problem in which CTFs that will give a best match to the corresponding displacement field are computed. By assuming that the gel is thick enough (e.g., >100 μm) to behave like an elastic half space, the application of the analytical Boussinesq solution obtains the discrete loads as an estimate of the traction forces. This can be achieved either by applying an inverse Fourier transform [57] or by solving a general regularized inverse problem [58]. The latter approach is more complicated, but has the advantage of allowing for the incorporation of *a priori* information such as levels of errors. A new CTFM approach applies effective pattern recognition algorithms in combination with finite element method (FEM) [60]. In this approach, a feature registration scheme is devised to match individual beads from the image pair. Furthermore, the gel is modeled in FEM as a 3-D object with its actual thickness using brick elements. By applying static condensation, FEM obtains CTFs in the form of forces on the surface nodes that lie within the boundary of the cells. This approach ensures reliable displacement calculation and enables simultaneous determination of CTFs of multiple cells.

Based on fluorescence microscopy imaging and computational mechanics, CTFM offers unique advantages over other CTF-sensing techniques in that it can sense and quantify CTFs of a wide range of individual cells or cell aggregates reliably, accurately, and efficiently. For example, the substrate used in CTFM, polyacrylamide gel (PAG), can be easily prepared and is linearly elastic in response to a wide range of stresses, and the deformation is completely recovered upon removal of the stress [61,62]. The stiffness of PAG can be easily adjusted in a broad range from as low as 10 Pa up to as high as 40 kPa [63]. In contrast, similar continuum substrate-based techniques using silicone membranes have been used less frequently, partially because it needs very delicate skill to fabricate ultra-thin membranes that are compliant enough for traction force measurement [4,49,51]. Similarly, a fluorescence resonance energy transfer (FRET)-based technique, which relies on the molecular packing of two fluorescently labeled peptides to indicate matrix deformation, also has only limited applications due to the technical complexity to determine CTFs quantitatively and the difficulty in preparing an optimal substrate containing two fluorophores, despite the fact that it can directly “visualize” the CTFs of a cell by monitoring changes in fluorescent colors [64].

## 3. Conclusions and Perspectives

Cells use CTFs, the mechanical forces that are generated by cells and transmitted to ECM, to probe their physical environment and to migrate, maintain their shape, and organize the ECM. Therefore, CTFs play a critical role in many fundamental biological processes including embryogenesis, tissue morphogenesis, angiogenesis, inflammation, wound healing, and metastasis. In addition, because perturbation of CTF-related proteins, such as actin, myosin, and focal adhesion proteins (e.g., integrins), of individual cells likely causes changes in CTFs, CTF may be used as a biophysical marker for characterizing phenotypic changes of cells in response to biochemical and biomechanical stimuli.

To date, a variety of techniques have been developed for sensing and quantifying CTFs of cells, either in a cell population as a whole or in individual cells. Among them, MFSA and CTFM represent the most advanced technologies and have since been widely used in CTF measurement. In addition to simple force measurement, such techniques also can be adopted to construct useful *in vitro* models that may aid in early diagnosis of diseases and screening for cellular and molecular therapies.

While CTF-sensing technologies are effective in many current situations, further advancement in these technologies and their applications should focus on the following aspects. First, improvement in the spatial resolution of force-sensing through both experimental and computational approaches is necessary. The ability to sense CTFs at nano-scale will greatly help bridge and integrate existing knowledge of biomechanics at molecular and cellular levels, respectively. Second, current sensing techniques for CTF measurement are mainly limited to 2-D environments, which are less physiological since cells reside in 3-D tissues *in vivo*. While attempts have been made in this regard recently [65,66], it is far from satisfactory because such measurements did not truly determine CTFs in a 3-D fashion. Thus, innovative sensing techniques are needed to enable sensing of CTFs in 3-D matrices such that the measured CTFs are more physiologically relevant. Findings from such studies will significantly advance our understanding of cell contractility in relation to normal and disease states. Third, in addition to improved resolution with nano-scale CTF-sensing capability, techniques that enable real time assays and/or combine with molecular biology techniques such as fluorescent protein fusion [49] should be used more extensively to decipher in more depth the molecular mechanisms and dynamics of CTF generation and transmission.
